# Design Technique and Efficient Polyphase Implementation for 2D Elliptically Shaped FIR Filters

**DOI:** 10.3390/s25154644

**Published:** 2025-07-26

**Authors:** Doru Florin Chiper, Radu Matei

**Affiliations:** 1 Faculty of Electronics, Telecommunications and Information Technology, “Gheorghe Asachi” Technical University of Iasi, 700506 Iasi, Romania; chiper@etti.tuiasi.ro; 2 Technical Sciences Academy of Romania—ASTR, 700050 Iaşi, Romania; 3 Academy of Romanian Scientists—AOSR, 030167 Bucharest, Romania; 4 Institute of Computer Science, Romanian Academy—Iaşi Branch, 700481 Iaşi, Romania

**Keywords:** 2D FIR filters, circular filters, analytical design, approximations, filter banks, polyphase decomposition, block filters

## Abstract

This paper presents a novel analytical approach for the efficient design of a particular class of 2D FIR filters, having a frequency response with an elliptically shaped support in the frequency plane. The filter design is based on a Gaussian shaped prototype filter, which is frequently used in signal and image processing. In order to express the Gaussian prototype frequency response as a trigonometric polynomial, we developed it into a Fourier series up to a specified order, given by the imposed approximation precision. We determined analytically a 1D to 2D frequency transformation, which was applied to the factored frequency response of the prototype, yielding directly the factored frequency response of a directional, elliptically shaped 2D filter, with specified selectivity and an orientation angle. The designed filters have accurate shapes and negligible distortions. We also designed a 2D uniform filter bank of elliptical filters, which was then applied in decomposing a test image into sub-band images, thus proving its usefulness as an analysis filter bank. Then, the original image was accurately reconstructed from its sub-band images. Very selective directional elliptical filters can be used in efficiently extracting straight lines with specified orientations from images, as shown in simulation examples. A computationally efficient implementation at the system level was also discussed, based on a polyphase and block filtering approach. The proposed implementation is illustrated for a smaller size of the filter kernel and input image and is shown to have reduced computational complexity due to its parallel structure, being much more arithmetically efficient compared not only to the direct filtering approach but also with the most recent similar implementations.

## 1. Introduction

In recent years, the technology, architecture and dedicated image processing algorithms for modern image sensors have known a spectacular development. For example, high-resolution image sensors for remote sensing applications must yield clear, low-noise images, mostly in visible but also in infrared or microwave domains. In order to provide the required high quality, relevant and precise information, images acquired by such sensors must undergo pre-processing stages using various enhancement and restoration operations. In this wide range of image processing tasks, digital filters and filter banks play a fundamental role, for instance, in analyzing images and decomposing them in a set of sub-band images, extracting relevant features and details. They are also extensively used in automotive, where high-speed image processing and feature extraction is highly demanded in real-time applications, such as driver assistance systems, etc.

Two-dimensional filters have been extensively investigated by many researchers in the vast field of digital signal and image processing due to their important applications in image processing, and a wide range of design techniques have been developed [1]. Unlike global numerical optimization algorithms, analytical design methods are based on 1D prototypes with imposed shapes and parameters, to which frequency transformations are applied, leading directly to the desired 2D filters. Perhaps the major benefit of the analytical approach is that the factored frequency response results, and the designed 2D filter is parametric and therefore easily adjustable.

There exists in the literature a wide variety of 2D filters, of the FIR or IIR type, with various shapes, each of them having applications in image processing tasks. One of the most currently used methods used in the analytical design of FIR filters is the McClellan transform [2,3]. Some important works in the field propose various computationally efficient 2D FIR filter structures also using frequency transformations, like multiplier-less circularly symmetric FIR filters using harmony search algorithms [4], sampling-kernel based interpolation [5], a design based on Gaussian approximation [6], and a single-parameter tunable 2-D Farrow structure [7]. Various transformations for the design of FIR and IIR filters are given in [8], and the efficient synthesis of 2D FIR filters with improved selectivity is approached in [9]. Two-dimensional filter banks of various types and shapes are widely used in essential applications, like texture analysis and segmentation, or feature extraction tasks. They decompose the frequency spectrum of the image into several sub-bands, which is required in coding applications, etc.

Different types of two-dimensional filter banks have been investigated [10], in particular directional [11] and nonuniform frequency plane partitioning [12]. An application of directional filter banks in fingerprints analysis is given in [13]. Elliptically shaped 2D filters, including directional ones, were approached in early papers such as [14,15]. A 2D wavelet filter bank with elliptical support was applied in iris recognition [16] and directional elliptical filters were used in robust human pose detection [17]. The design of 2D FIR filters with a circular and elliptical shape based on a swarm optimization algorithm is proposed in [18].

Gaussian separable filtering can also be combined with other methods, such as integral image techniques to construct a Gaussian pyramid following an SIFT algorithm; it may be followed, for instance, by Canny edge detection and dilation operations in order to obtain an optimized edge detection, with superior performances and reduced complexity, also suitable for real-time processing [19]. Such analytical design methods could also be extended to 3D image processing, with interesting applications in physics. For instance, paper [20] presents a 3D digital image correlation approach for accurately measuring strain and deformations in vinyl chloride-coated metal multilayer sheets.

Some computationally efficient structures of 2D FIR filters using block filtering, systolic arrays, etc., are elaborated on in [21,22].

Several analytical design techniques for various 2D filters have also been proposed in previous works. Thus, we studied a class of 2D FIR circular filter banks and their efficient implementation using a polyphase approach in [23], the design and applications of adjustable elliptical and circular 2D IIR filters in [24] and another type of directional elliptically shaped IIR filter, with applications in the detection of oriented straight lines and other details in images, in [25].

In this paper, an analytical design procedure is elaborated on for a particular class of 2D filter, specifically 2D FIR Gaussian elliptical filters. A uniform elliptical 2D filter bank is also designed, with a specified number of component filters, based on a corresponding 1D prototype with a Gaussian frequency response. This is easily approximated using Fourier series expansion. By simply shifting the low-pass filter to equally spaced peak frequencies, we also obtain band-pass filters, thus generating a uniform filter bank. We have determined a particular 1D to 2D frequency transformation, which is applied to the 1D prototype and yields directly the desired 2D filter with elliptically shaped support. The 2D filters result in accurate shapes and negligible distortions. The designed elliptical FB was tested on a greyscale image, which was decomposed into sub-band components, and then the original image was reconstructed very accurately from these components. In a particular case, very selective low-pass directional filters can detect and extract straight lines with specified orientation from images, as shown by the simulation results.

A novel and more efficient implementation solution is also proposed for the designed 2D FIR filter. It has a significantly lower complexity than a recent implementation in [21] while maintaining the same throughput. The proposed solution is based on a new algorithm for 2D FIR filtering with a smaller kernel of 3 × 3, which has been extended at a larger kernel size of 30 × 30 using a polyphase decomposition of the given 2D filtering operation and block filtering. In order to facilitate the reformulation of the algorithm for 2D FIR filtering for a polyphase implementation, we have introduced some auxiliary vectors. Thus, it was possible to extend the proposed 2D filtering algorithm for a significantly larger size.

This paper is organized as follows. Section 2 presents the analytical design technique, first approximating a Gaussian prototype using Fourier series, then generating an uniform Gaussian filter bank with a specified number of components, peak frequencies and bandwidths. Next, a specific 1D to 2D frequency transformation is determined, which will be applied to the given prototype and will lead directly to the desired elliptically shaped 2D filter with a specified orientation in the frequency plane. Next, several design examples are given, showing the 2D filter characteristics (the frequency response and contour plot), for the components of an elliptical filter bank, and then for very selective filters with various orientations. In Section 3, we present an example of image analysis and reconstruction using the elliptical filter bank by decomposing it into sub-band images. A simulation of straight line extraction from a test image, using narrow directional elliptical filters, is also given. The proposed filtering implementation at the system level, based on the polyphase and block structure, is described in Section 4. Some comparative discussions regarding the filter design and computational complexity of the proposed implementation are provided in Section 5. Finally, conclusions are given in the last section.

## 2. Analytical Design Technique for 2D Elliptical FIR Filters

In this main section, a novel analytical design procedure for a class of 2D FIR filters with elliptically shaped frequency response is presented. The proposed technique is based on a suitable 1D prototype with imposed specifications (peak frequency and bandwidth), to which 1D to 2D frequency mapping is applied, which directly leads to the desired 2D filter. We have chosen as a prototype a Gaussian-shaped filter, which has the useful property of scalability on the frequency axis. A uniform filter bank prototype is first derived, which yields a 2D elliptically shaped filter bank through the elaborated frequency mapping.

### 2.1. Design of Gaussian FIR Filter Prototype Based on Fourier Series Approximation

As is well known, the Gaussian filter function in the frequency domain can be expressed as $G\left(\omega \right)=\exp\left(-\sigma^{2}\omega^{2}/2\right)$, where parameter σ represents variance. In order to derive a simpler, more practical form, we will apply the substitution $p=\sigma^{2}/2$, otherwise written as $\sigma =\sqrt{2p}$. In this way, the low-pass filter with a Gaussian shape will be expressed in the more convenient form $G_{LP}\left(\omega \right)=\exp\left(-p\cdot \omega^{2}\right)$. We consider that the Gaussian filter is defined by the range [−π,π] as its support by extending it on the entire frequency axis as a periodic function with period 2π. We derive the following approximation through a trigonometric polynomial, by developing the Gaussian $G_{LP}\left(\omega \right)$ in Fourier series, up to a given order, *N*:

(1) $$G_{LP}\left(\omega \right)=\exp\left(-p\cdot \omega^{2}\right)≅\frac{1}{2\sqrt{p\pi }}\cdot \left(1+2\cdot \sum_{n=1}^{N}\exp\left(-\frac{n^{2}}{4p}\right)\cdot \cos n\omega \right)=H_{LP}\left(\omega \right)$$

From this expression of the Gaussian low-pass prototype, we also obtain a band-pass (BP) prototype by shifting this low-pass filter to the symmetric frequencies $\pm \omega_{0}$:

(2) $$\begin{array}{c} H_{BP}\left(\omega \right)=H_{LP}\left(\omega -\omega_{0}\right)+H_{LP}\left(\omega +\omega_{0}\right)=\exp\left(-p\cdot {\left(\omega -\omega_{0}\right)}^{2}\right)+\exp\left(-p\cdot {\left(\omega +\omega_{0}\right)}^{2}\right)≅\\ \frac{1}{\sqrt{p\pi }}\cdot \left(1+2\cdot \sum_{n=1}^{N}\exp\left(-\frac{n^{2}}{4p}\right)\cdot \cos\;(n\omega_{0})\cdot \cos n\omega \right) \end{array}$$

Expressions (1) and (2) above can easily be calculated in a MATLAB routine. Next, the low-pass, band-pass and high-pass components of the specified FIR filter bank prototype will be derived.

### 2.2. The Design of a Gaussian Uniform FIR Filter Bank Prototype

In this section, a uniform, Gaussian filter bank (FB) prototype with seven-component filters will be designed, consisting in one low-pass (LP) filter, five band-pass (BP) filters and one high-pass (HP) filter.

As in every uniform filter bank, the peak frequencies are equally spaced on the frequency axis. For the 5 BP filters, we impose a bandwidth equal to B=π/6, while the LP and HP filters have each half of this bandwidth, namely B/2=π/12. The *k*-th ideal Gaussian band-pass filter is derived by shifting the low-pass prototype to the frequency $\omega_{0,k}=k\cdot \omega_{0}$, and its frequency response has the expression

(3) $$G_{BP\;k}\left(\omega \right)=G_{LP}\left(\omega -k\omega_{0}\right)+G_{LP}\left(\omega +k\omega_{0}\right)=\exp\left(-p\cdot {\left(\omega -k\omega_{0}\right)}^{2}\right)+\exp\left(-p\cdot {\left(\omega +k\omega_{0}\right)}^{2}\right)$$

**Figure 1:**
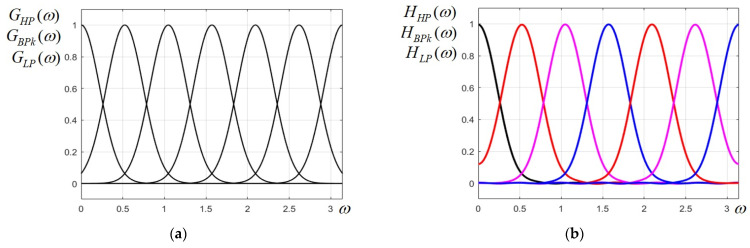
(**a**) Ideal Gaussian uniform FB prototype; (**b**) designed Gaussian uniform FB prototype for 2D FB.

Next, scaling parameter *p* has to be calculated for the specified bandwidth value. For our purpose, the filter bandwidth is defined at 0.5 of the peak value (at 6 dB). The magnitude characteristics of any two consecutive, adjacent filters partially overlap and intersect at the value 0.5. Considering the LP filter given by $G_{LP}\left(\omega \right)=\exp\left(-p\cdot \omega^{2}\right)$, we apply the condition $G_{LP}\left(B/2\right)=\exp\left(-p\cdot B^{2}/4\right)=0.5$, also written $\exp\left(p\cdot B^{2}/4\right)=2$; thus, the value of scaling parameter *p* is $p=4\ln2/B^{2}$. Since the bandwidth of each component filter is B=π/6, we obtain the scaling parameter value $p=144\cdot \ln2/\pi^{2}≅10.1132$. The ideal uniform Gaussian filter bank is plotted in Figure 1a. For a very accurate approximation and a small ripple level, the Fourier series given by (1) and (2) was truncated at a number of *N* = 12 terms. The approximation order must be a trade-off between a small distortion level and a reasonable size of the filter matrices for a convenient implementation.

In the next step, the components of the uniform prototype filter bank are determined. Following the steps of the design method described above, knowing the bandwidths and peak frequencies of component filters, using Equations (1) and (2), the frequency responses of the FB components are calculated.

For instance, the LP Gaussian prototype, approximated by a truncated Fourier series using (1), results in

(4) $$\begin{array}{l} H_{LP}\left(\omega \right)=0.0887063+0.173081\cdot \cos\omega +0.1607092\cdot \cos2\omega +0.1420237\cdot \cos3\omega +0.119456\cdot \cos4\omega \\ +0.095628\cdot \cos5\omega +0.0728597\cdot \cos6\omega +0.052834\cdot \cos7\omega +0.036465\cdot \cos8\omega +0.0239533\cdot \cos9\omega \\ +0.0149755\cdot \cos10\omega +0.008911\cdot \cos11\omega +0.00504655\cdot \cos12\omega \end{array}$$

Using trigonometric identities, this is further written as follows:

(5) $$\begin{array}{r} H_{LP}\left(\omega \right)=10.3353344\cdot \left(\cos\omega +0.9923\right)\left(\cos\omega +0.931466\right)\left(\cos\omega +0.81361\right)\left(\cos\omega +0.646128\right)\\ \cdot \left(\cos\omega +0.4395183\right)\left(\cos\omega +0.206737\right)\left(\cos\omega -0.03766\right)\left(\cos\omega -0.278484\right)\\ \cdot \left(\cos\omega -0.501253\right)\left(\cos\omega -0.676875\right)\left({(\cos\omega )}^{2}-1.652607\cdot \cos\omega +0.695704\right) \end{array}$$

Next, the frequency responses of the BP filter components of the bank are given as

(6) $$\begin{array}{l} H_{BP1}\left(\omega \right)=20.6719213\cdot (\cos\;\omega +0.99237)(\cos\;\omega +0.93206)(\cos\;\omega +0.815269)(\cos\;\omega +0.649373)\\ \cdot (\cos\;\omega +0.444886)(\cos\;\omega +0.2148096)(\cos\;\omega -0.026717)(\cos\;\omega -0.242526)(\cos\;\omega -1.009604)\\ \cdot (\cos\;\omega -1.11825)((\cos\;\omega {)}^{2}-0.887092\cdot \cos\;\omega +0.228733) \end{array}$$

(7) $$\begin{array}{r} H_{BP2}\left(\omega \right)=20.67192\cdot (\cos\;\omega +0.992623)(\cos\;\omega +0.934318)(\cos\;\omega +0.82153)(\cos\;\omega +0.661733)\\ \cdot (\cos\;\omega +0.464866)(\cos\;\omega +0.273917)(\cos\;\omega -0.983609)(\cos\;\omega -0.998796)\\ \cdot \left({(\cos\;\omega )}^{2}+0.17237\cdot \cos\omega +0.0519616\right)\left({(\cos\omega )}^{2}-1.897523\cdot \cos\omega +0.90847\right) \end{array}$$

(8) $$\begin{array}{r} H_{BP3}\left(\omega \right)=20.67192\cdot (\cos\;\omega +0.993351)(\cos\;\omega +0.9408166)(\cos\;\omega +0.838538)(\cos\;\omega +0.722343)\\ \cdot (\cos\;\omega -0.722343)(\cos\;\omega -0.838538)(\cos\;\omega -0.9408166)(\cos\;\omega -0.993351)\\ \cdot {(\cos\;\omega )}^{2}+1.191947\cdot \cos\omega +0.387847){\left((\cos\;\omega \right)}^{2}-1.191947\cdot \cos\;\omega +0.387847) \end{array}$$

(9) $$\begin{array}{r} H_{BP4}\left(\omega \right)=20.67192\cdot (\cos\;\omega -0.992623)(\cos\;\omega -0.934318)(\cos\;\omega -0.82153)(\cos\;\omega -0.661733)\\ \cdot (\cos\;\omega -0.464866)(\cos\;\omega -0.273917)(\cos\;\omega +0.983609)(\cos\;\omega +0.998796)\\ \cdot \left({(\cos\;\omega )}^{2}-0.17237\cdot \cos\;\omega +0.0519616\right)\left({(\cos\;\omega )}^{2}+1.897523\cdot \cos\;\omega +0.90847\right) \end{array}$$

(10) $$\begin{array}{l} H_{BP5}\left(\omega \right)=20.6719213\cdot (\cos\;\omega -0.99237)(\cos\;\omega -0.93206)(\cos\;\omega -0.815269)(\cos\;\omega -0.649373)\\ \cdot (\cos\;\omega -0.444886)(\cos\;\omega -0.2148096)(\cos\;\omega +0.026717)(\cos\;\omega +0.242526)(\cos\;\omega +1.009604)\\ \cdot (\cos\;\omega +1.11825)((\cos\;\omega {)}^{2}+0.887092\cdot \cos\;\omega +0.228733) \end{array}$$

(11) $$\begin{array}{r} H_{HP}\left(\omega \right)=10.3353344\cdot \left(\cos\omega -0.9923\right)\left(\cos\omega -0.931466\right)\left(\cos\omega -0.81361\right)\left(\cos\omega -0.646128\right)\\ \cdot \left(\cos\omega -0.4395183\right)\left(\cos\omega -0.206737\right)\left(\cos\omega +0.03766\right)\left(\cos\omega +0.278484\right)\\ \cdot \left(\cos\omega +0.501253\right)\left(\cos\omega +0.676875\right)\left({(\cos\omega )}^{2}+1.652607\cdot \cos\omega +0.695704\right) \end{array}$$

As can be noticed from the expressions of frequency responses given by (5)–(11), the filters of the prototype filter bank with symmetric peak frequencies with respect to the value ω=π/2 (at the middle of the interval) have symmetric zeros, which is according to digital filter theory. For instance, the zeros of the fifth BP filter are the zeros of the first BP filter with a changed sign, the second and forth BP filter also have opposite zeros, etc. As the FB is made up of an odd number of filters, the middle (third) BP filter, with peak frequency $\omega_{0}=\pi /2$, has no pair, and its transfer function has pairs of complementary zeros. The uniform Gaussian prototype filter bank designed using the above procedure is plotted in Figure 1b and it looks very similar to its ideal counterpart in Figure 1a, with a very low level of ripple. In Table 1, the maximum ripple in the stop-band for all the filters of the filter bank are given. Due to the Gaussian shape, these filters have no ripple in the pass-band.

### 2.3. Design of 2D FIR Filters with Elliptically Shaped Frequency Response Using Frequency Transformation

In this work, we approached the analytical design of a particular class of anisotropic (directional) filters, with elliptically shaped support in the frequency plane. The shape of their characteristics is determined by specifying the values of the major and minor semi-axes of the ellipse (E and F). Their orientation is given by angle φ, formed by its longitudinal axis with the $\omega_{\;1}$-axis of the frequency plane. In the following paragraphs, we determine the expression of the 1D to 2D frequency transformation that leads to 2D elliptically shaped filters.

According to the proposed analytical design technique, a 2D filter with an elliptically shaped frequency response is obtained by applying to the specified 1D prototype the following 1D to 2D frequency transformation $\omega^{2}\to S_{\varphi }(\omega_{1},\omega_{2})$, with the expression

(12) $$\begin{array}{c} \omega^{2}\to S_{\varphi }(\omega_{1},\omega_{2})=\omega_{1}^{2}\left(\frac{\cos^{2}\varphi }{E^{2}}+\frac{\sin^{2}\varphi }{F^{2}}\right)+\omega_{2}^{2}\left(\frac{\sin^{2}\varphi }{E^{2}}+\frac{\cos^{2}\varphi }{F^{2}}\right)+\omega_{1}\omega_{2}\sin(2\varphi )\left(\frac{1}{F^{2}}-\frac{1}{E^{2}}\right)\\ \;=a_{0}\cdot \omega_{1}^{2}+b_{0}\cdot \omega_{2}^{2}+c_{0}\cdot \omega_{1}\omega_{2} \end{array}$$

In the above mapping, *E* and *F* are the values of major and minor semi-axes of the ellipse, respectively. The expressions of the coefficients $a_{0}$, $b_{0}$ and $c_{0}$ are found by direct identification. In our case, applying this frequency mapping to a Gaussian filter with the specified peak frequency and bandwidth, we derive a 2D filter with elliptically shaped support in the frequency plane, depending on the parameters *E*, *F* and φ. These define the aspect of the ellipse and its orientation through the mapping (12).

A more convenient form of the frequency mapping (12) results using the simple identity $\omega_{\;1}\omega_{\;2}=0.5\cdot \left({\left(\omega_{\;1}+\omega_{\;1}\right)}^{2}-\omega_{\;1}^{2}-\omega_{\;2}^{2}\right)$; making this substitution in (12) and re-arranging, we obtain

(13) $$\omega^{2}\to S_{\varphi }(\omega_{1},\omega_{2})=a\cdot \omega_{1}^{2}+b\cdot \omega_{2}^{2}+c\cdot {\left(\omega_{1}+\omega_{2}\right)}^{2}$$

Next, introducing the notations $q=1/E^{2}+1/F^{2}$, $r=1/E^{2}-1/F^{2}$, we derive the following expressions of the coefficients *a*, *b* and *c*:

(14) $$a=a_{0}-0.5\;c_{0}=q+r\cdot \cos(2\varphi )+r\cdot \sin(2\varphi )$$

(15) $$b=b_{0}-0.5\;c_{0}=q-r\cdot \cos(2\varphi )+r\cdot \sin(2\varphi )$$

(16) $$c=0.5\;c_{0}=-r\cdot \sin(2\varphi )$$

Applying the general 1D to 2D mapping (13) to the ideal Gaussian prototype $H_{P}(\omega )=\exp\;(-p\omega^{2})$, we obtain the ideal 2D frequency response:

(17) $$H_{E}(\omega_{\;1},\omega_{2})=\exp\left(-p\cdot \left(a\cdot \omega_{1}^{2}+b\cdot \omega_{2}^{2}+c\cdot {\left(\omega_{1}+\omega_{2}\right)}^{2}\right)\right)$$

The Gaussian 1D prototype, with specified selectivity, was approximated as a factored trigonometric polynomial in variable cosω as in Relations (5)–(11). We will next determine the following frequency mapping, which is derived from (13):

(18) $$\cos\omega \to \cos\sqrt{a\cdot \omega_{1}^{2}+b\cdot \omega_{2}^{2}+c\cdot {\left(\omega_{1}+\omega_{2}\right)}^{2}}=\cos\sqrt{S_{\varphi }(\omega_{1},\omega_{2})}$$

The final purpose is to find a 1D to 2D mapping of the form $\cos\omega \to F(z_{1},z_{2})$, which can lead directly to the transfer function of a 2D digital filter with the desired shape.

In this step, the efficient Chebyshev series approximation is used, since it has the major advantage of yielding a precise and uniform approximation of a given function on a specified range of values. Using the symbolic calculation software MAPLE 6.02 and an intermediate change in variable, we derive the following accurate approximation (for ω>0, specifically ω∈[0,π]):

(19) $$\cos\sqrt{\omega }≅0.996696-0.486389\cdot \omega +0.0332286\cdot \omega^{2}$$

**Figure 2:**
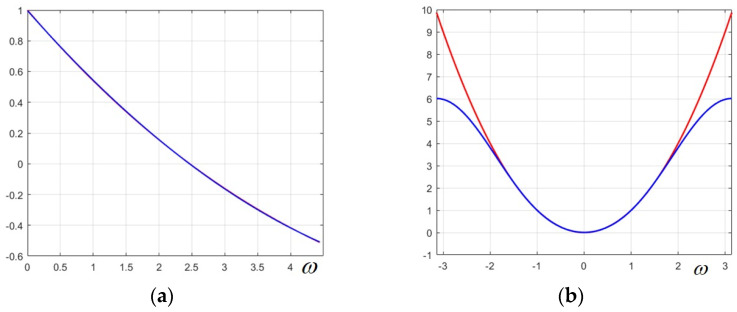
(**a**) The function $\cos\sqrt{\omega }$ (plotted in red) and its approximation (in blue)—they are practically superposed; (**b**) the function $\omega^{2}$ (in red) and its trigonometric approximation using Chebyshev series (in blue).

This approximation is plotted in Figure 2a in blue and is practically superposed on the exact function $\cos\sqrt{\omega }$, which shows that the approximation is very precise.

Considering Equation (18) as well, we obtain

(20) $$\cos\sqrt{S_{\varphi }(\omega_{1},\omega_{2})}≅0.996696-0.486389\cdot S_{\varphi }(\omega_{1},\omega_{2})+0.0332286\cdot S_{\varphi }^{2}(\omega_{1},\omega_{2})$$

Using again the Chebyshev series method, the following low-order trigonometric approximation for the function $\omega^{2}$ results in

(21) $$\omega^{2}≅2.734863-3.003487\cdot \cos\omega +0.274406\cdot \cos2\omega$$

This approximation is plotted as the blue curve in Figure 2b, while the exact function $\omega^{2}$ is plotted in red. It can be noticed that the approximation is very accurate at least on the interval [−2,2], and it tends to diverge visibly towards the margins of the interval.

Regarding this marginal error, even if it is large towards the limits of the interval, it does not affect the designed filters much since the component filters of the bank are derived from a narrow-band low-pass filter, and the marginal errors cancel out. Therefore, it is essential that the approximation is very precise on a sufficiently large interval around zero, as the plot in Figure 2b shows.

The above approximation is then written separately for the frequency variables $\omega_{1}$ and $\omega_{\;2}$ and their sum $\omega_{1}+\omega_{2}$ as follows:

(22) $$\omega_{1}^{2}≅2.734863-3.003487\cdot \cos\omega_{1}+0.274406\cdot \cos2\omega_{1}$$

(23) $$\omega_{2}^{2}≅2.734863-3.003487\cdot \cos\omega_{2}+0.274406\cdot \cos2\omega_{2}$$

(24) $${\left(\omega_{1}+\omega_{2}\right)}^{2}≅2.734863-3.003487\cdot \cos(\omega_{1}+\omega_{2})+0.274406\cdot \cos2(\omega_{1}+\omega_{2})$$

The next design step is to substitute Relations (22)–(24) into (13), obtaining an expression of $S_{\varphi }(\omega_{1},\omega_{2})$ which is then introduced in (20); finally, a trigonometric expression of the function $\cos\sqrt{S_{\varphi }(\omega_{1},\omega_{2})}$ in the variables $\cos\omega_{\;1}$ and $\cos\omega_{\;2}$ is determined. Considering this two-variable function as a 2D discrete Fourier transform, by directly identifying the corresponding coefficients, we finally reach a matrix of size 5×5, with the general form, symmetric with respect to the central element:

(25) $$S_{\varphi }=\left[\begin{array}{ccccc} 0 & 0 & \alpha_{5} & 0 & \alpha_{7}\\ 0 & 0 & \alpha_{3} & \alpha_{4} & 0\\ \alpha_{6} & \alpha_{2} & \alpha_{1} & \alpha_{2} & \alpha_{6}\\ 0 & \alpha_{4} & \alpha_{3} & 0 & 0\\ \alpha_{7} & 0 & \alpha_{5} & 0 & 0 \end{array}\right]$$

where the matrix elements have the following expressions, which depend on the ellipse semi-axes parameters *E* and *F* and on the angle of orientation, φ:

(26) $$\alpha_{1}=5.469726\cdot q+2.734863\cdot r\cdot \sin2\varphi$$

(27) $$\alpha_{2}=-1.501744\cdot q-1.501744\cdot r\cdot (\sin2\varphi +\cos2\varphi )$$

(28) $$\alpha_{3}=-1.501744\cdot q+1.501744\cdot r\cdot (cos2\varphi -\sin2\varphi )$$

(29) $$\alpha_{4}=1.501744\cdot r\cdot \sin2\varphi$$

(30) $$\alpha_{5}=0.137203\cdot q+0.137203\cdot r\cdot (sin2\varphi -\cos2\varphi )$$

(31) $$\alpha_{6}=0.137203\cdot q+0.137203\cdot r\cdot (sin2\varphi +\cos2\varphi )$$

(32) $$\alpha_{7}=-0.137203\cdot r\cdot \sin2\varphi$$

Because the parametric matrix, $S_{\varphi }$, in (25) corresponds to the function $S_{\varphi }(\omega_{1},\omega_{2})$, then taking into account Equation (20), the function $\cos\sqrt{S_{\varphi }(\omega_{1},\omega_{2})}$ will in turn correspond to matrix $S_{E\varphi }$ of size 9×9, according to the following relation:

(33) $$S_{E\varphi }≅0.996696\cdot S_{0}-0.486389\cdot S_{\varphi 1}+0.0332286\cdot S_{\varphi }*S_{\varphi }$$

where by the symbol ∗, we denote the matrix convolution operation. In the above relation, $S_{0}$ is a 9×9 null matrix, with a central element of value one. The matrix $S_{\varphi 1}(9\times 9)$ is obtained by bordering $S_{\varphi }$ (5×5) with zero values up to size 9×9.

Taking into account Expressions (5)–(11) for the factored frequency responses of the 1D prototype filter bank components, we can express them in the following form, which is, in general, a product of first-order and second-order factors:

(34) $$H_{P}(\omega )=k\cdot \prod_{i=1}^{n}\left(\cos\omega +b_{i}\right)\cdot \prod_{j=1}^{m}\left({\left(\cos\omega \right)}^{2}+b_{1j}\cdot \cos\omega +b_{2j}\right)$$

In (34), the coefficients $b_{i}$, $b_{1,j}$ and $b_{2,j}$ are generic coefficients which result in each factor in the prototype frequency response $H_{P}(\omega )$, while *k* is the constant resulting in front.

In the next step, simply by substituting in each factor of the general frequency response (34) the variable cosω with the function $\cos\sqrt{S_{\varphi }(\omega_{1},\omega_{2})}$ derived as before, we find directly the factored frequency response of any 2D elliptically filter, generally denoted as $H_{E}(\omega_{1},\omega_{2})$ and containing both first-order and second-order factors:

(35) $$H_{E}(\omega_{1},\omega_{2})=k\cdot \prod_{i=1}^{n}\left(S+b_{i}\right)\cdot \prod_{j=1}^{m}\left(S^{2}+b_{1j}\cdot S+b_{2j}\right)$$

where *S* is a concise notation for the two-variable function $S_{\varphi }(\omega_{1},\omega_{2})$. Thus, the large kernel H corresponding to the filter function $H_{E}(\omega_{1},\omega_{2})$ can be expressed directly as a convolution product of smaller matrices, corresponding to each factor from (35):

(36) $$H=k\cdot (S_{1}*\ldots *S_{i}*\ldots *S_{n})*(P_{1}*\ldots *P_{j}*\ldots *P_{m})$$

Matrix Expression (36) corresponds to the factored frequency response (35). Using matrix $S_{E\varphi }$ of size 9×9 given in (25) and taking into account Equations (22)–(24), each of the matrices $S_{i}$ from (36) is derived by adding coefficient $b_{i}$ occurring in first-order factors in (34) to the center element in matrix $S_{E\varphi }$. Matrix $P_{j}$, corresponding to second-order factors, is

(37) $$P_{j}=S_{E\varphi }*S_{E\varphi }+b_{1j}\cdot S_{E\varphi 1}+b_{2j}\cdot S_{E\varphi 0}$$

where $S_{E\varphi 0}$ is a null matrix of size 17×17 with a central element of value one. Matrix $S_{E\varphi 1}\;(17\times 17)$ results from bordering matrix $S_{E\varphi }$ (size 9×9) with zeros, symmetrically on all sides; here, the symbol * denotes matrix convolution.

Another advantage of the proposed method is that it yields the 2D filter frequency response directly factored, which may be a major advantage in a cascade structure (sequential) implementation of these filters. Some design examples of 2D elliptical filters are presented as follows.

### 2.4. Design Examples of 2D FIR Filters with Elliptical Shape

**Figure 3:**
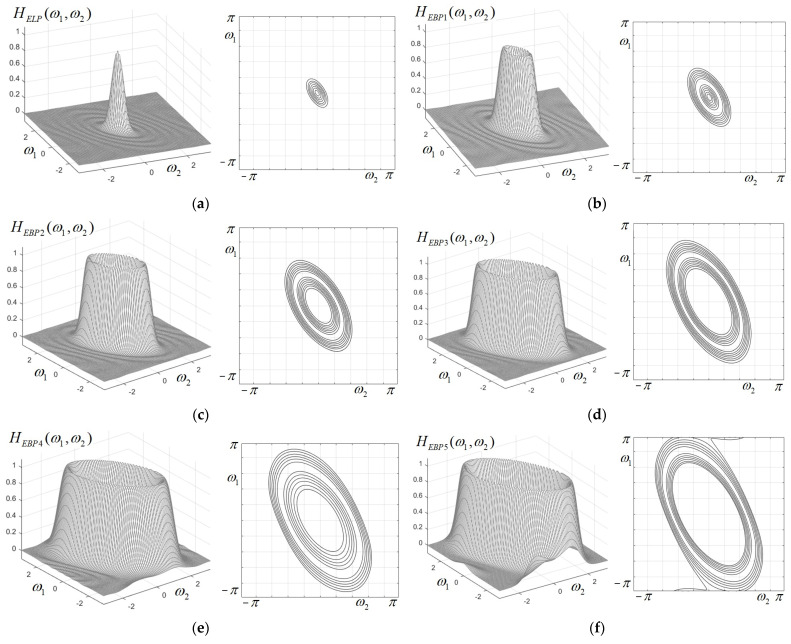
Frequency responses and corresponding contour plots for the component filters of the 2D elliptically shaped FIR filter bank, with the aspect ratio E/F=2 and orientation angle φ=π/6: (**a**) low-pass filter $H_{ELP}(\omega_{1},\omega_{2})$; (**b**–**f**) band-pass filters $H_{EBP_{k}}(\omega_{1},\omega_{2})$, with *k =* 1,…,5.

As a first design example using the proposed analytical technique, we have applied the frequency transformation determined above to each component of the prototype filter bank of a Gaussian shape, with the frequency responses given by Expressions (6)–(11). For the aspect ratio (between the semi-axes of the ellipse), we considered the value E/F=2 and the orientation angle of value φ=π/6. It can be noticed from Figure 3 that the resulting elliptical filters have a very accurate elliptical shape, steep transition and negligible ripple in the stop-band region.

**Figure 4:**
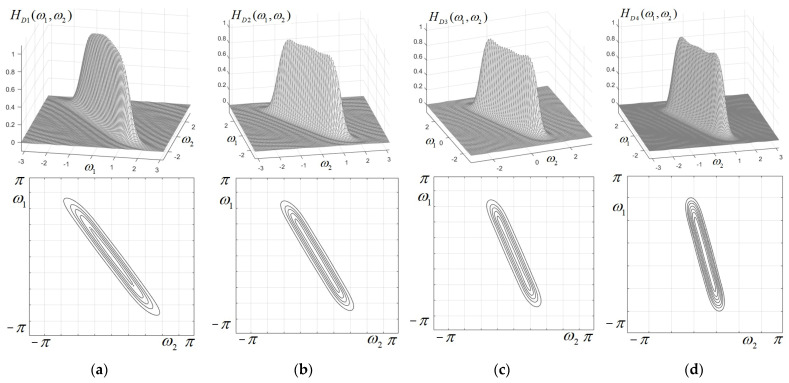
Frequency responses (**above**) and contour plots (**below**) for very selective directional low-pass elliptical filters with semi-axes values E=16 and F=1 and orientation angles: (**a**) φ=π/5; (**b**) φ=π/6; (**c**) φ=π/8; (**d**) φ=π/12.

If the aspect ratio of the ellipse (the ratio of semi-axes) E/F is given a larger value, a very stretched ellipse is obtained, and the corresponding 2D elliptically shaped filters will be very narrow, with a very selective directional characteristic. In Figure 4, the frequency responses and contour plots were displayed for four low-pass very selective directional filters. They have the semi-axes values E=16 and F=1 and four orientation angles, namely φ=π/5, φ=π/6, φ=π/8 and φ=π/12. As will be shown, such filters can be applied in detecting straight lines and other oriented objects in images.

The next section presents two important applications of the designed 2D elliptical filters, namely the analysis of a test image by the filter bank and the detection of oriented straight lines from an image, using very selective directional filters.

## 3. Applications and Simulation Results

This section presents a simulation example of the decomposition and reconstruction of a real-life test image using the designed elliptical uniform filter bank and a quantitative evaluation of the reconstruction quality, using specific measures. Then, a simulation example of directional filtering using very selective low-pass elliptical filters, with various orientation angles in the frequency plane, is given. This shows the capability of these narrow filters in extracting oriented straight lines from images.

### 3.1. Image Analysis Using the Designed Elliptical Uniform Filter Bank

**Figure 5:**
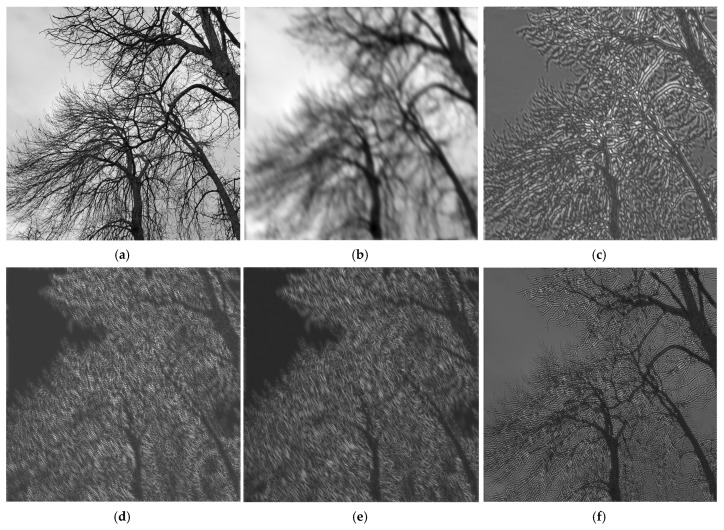

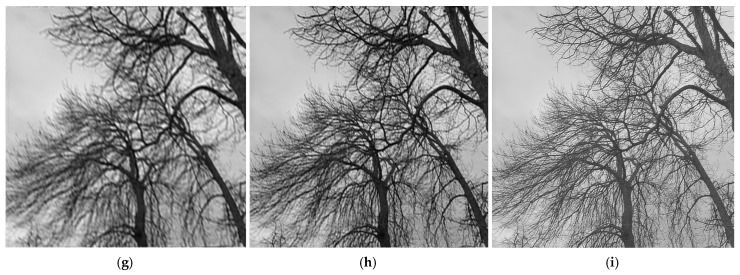
Image analysis using the elliptically shaped filter bank: (**a**) original “Trees_in_winter” image; (**b**) LP-filtered; (**c**–**f**) BP-filtered with BPF1, BPF2, BPF3, BPF4, respectively; (**g**,**h**) recovered image by summing first 2 and 3 components; (**i**) recovered image by summing all 7 components (sub-band images).

In this section, we show a useful application of the uniform elliptically shaped FIR filter bank designed through the proposed analytical technique. The following simulation will be performed on a grayscale test image of size 643×643 shown in Figure 5a, representing some branches of trees without foliage in winter time. This particular test image was chosen due to its structure of fine details, represented by the tree ramifications. The image was filtered by passing it through the six components of the filter bank (one low-pass filter and five band-pass filters). In this case, the elliptical uniform filter bank actually decomposes the test image into sub-band images, thus behaving as a usual analysis filter bank. The original image shown in Figure 5a is first low-pass filtered by the filter $H_{ELP}$, which has a relatively narrow bandwidth, both transversally and longitudinally, for the specified values of the ellipse semi-axes. Figure 5b showing the output of the LP filter is very blurred, and the fine details, like thin twigs, can be no longer distinguished. Next, Figure 5c–f showing the outputs of the first four band-pass filters contain specific details, with higher and higher frequencies, corresponding to the selected region from the frequency plane.

As in every synthesis filter bank, the original test image that was decomposed by the analysis FB can be reconstructed by adding the sub-band component images. For instance, (g) is created by summing the first two component (b) and (c). Next, (h) is obtained through the summation of the first three components, namely (b–d), and so on. It can be observed that even the partially reconstructed (h) has regained in some measure its finer details. By summing all the six-component images, the reconstructed (i) will be obtained. It can be noticed that (i) is visually, from a subjective point of view, quite close to the original image, as it displays even the finest details quite clearly. This proves experimentally that the designed elliptically shaped uniform filter bank practically behaves and can be used as analysis filter bank for decomposing images into sub-band components.

Next, a quantitative analysis of the quality of image decomposition and reconstruction is performed using specific measures.

### 3.2. Quantitative Evaluation of Filtering Performance Using Relative Energy, RMSE and PSNR

In order to evaluate the relevance of image decomposition using the designed elliptical uniform filter bank, we can also evaluate the energy of each component sub-band image using the well-known formula $E_{k}=\sqrt{\sum_{i=1}^{M}\sum_{j=1}^{N}p_{ij}}$, where the image is of size *M* × *N*. We will use the more useful expression of the relative energy as a percentage:

(38) $$E_{Rk}=\frac{100}{M\cdot N}\cdot \sqrt{\sum_{i=1}^{M}\sum_{j=1}^{N}p_{ij}}\;\left(\%\right)$$

**Figure 6:**
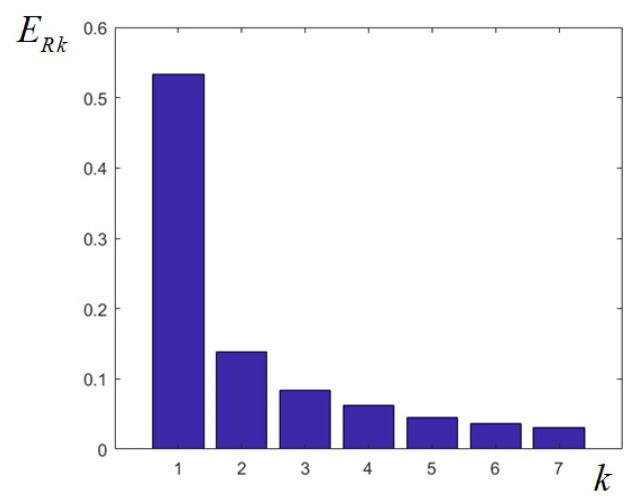
Relative energies calculated for the 7 images resulting from the output of the uniform elliptical filter bank.

Calculating the energies of the seven filtered images created as the output of the designed filter bank, the values given in Table 2 are obtained; summing these values, we find out that they add up to 93.13%. It can also be noticed that about 53% of the image energy is contained in the low-pass component (in the frequency domain around zero), while almost 82% is contained in the first four components at the output of LP filter and first three BP filters. As can be seen from the bar plot in Figure 6, the relative energies of the sub-band images decrease monotonically.

In this way, the decomposition of the test image into sub-band images can also be described in terms of image energy. For the designed elliptical filter bank, the monotonical decrease in the energies of the sub-band images, the highest energy corresponding to the LP filter, explains the fact that the original image can be gradually reconstructed by successively summing up the sub-band images from low to high frequencies and that the higher frequency components have the least effect on the recovered image.

The simulation results of the image reconstruction for its component sub-band images show that the original test image is recovered with more and more precision by adding up the components, from the low-pass component to the high-pass one. Even if visually the reconstructed images seem more and more similar to the original one, it would be useful to evaluate their performance in a quantitative manner as well, using some objective metrics commonly applied in image processing.

The most appropriate measures for our purpose would be the root mean squared error (RMSE) and, derived from it, the peak signal-to-noise ratio (PSNR), which is the ratio between the maximum value or power of a signal and the power of noise that affects the signal. In our case, the images are not actually affected by noise. Therefore, we consider as “noise” the difference or error between the intermediate reconstructed image and the original image. The larger the value of the PSNR, the more similar the reconstructed image is to the original image, and the smaller the reconstruction error is.

Because, usually, signals or images have a wide dynamic range, the PSNR is most commonly expressed as a logarithmic quantity in decibels (dB).

Given a grayscale image, I, unaffected by noise, and its noisy or distorted version, J, the RMSE is defined mathematically as

(39) $$RMSE=\sqrt{\frac{1}{M\times N}\cdot \sum_{i=0}^{M-1}\sum_{j=0}^{N-1}{\left[I(i,j)-J(i,j)\right]}^{2}}$$

where by I(i,j) and J(i,j) we denote the value of the current pixel (i,j) from the two images, respectively. Based on the root mean squared error, the PSNR (in decibels) is defined by the expression

(40) $$PSNR=20\cdot \log_{10}\left(Max(I)/RMSE\right)$$

In the above formula, Max(I) is the maximum possible pixel value of the image. In the most common situation, when pixels are represented on 8 bits, this value is 255. Thus, in calculating the RMSE and PSNR, noisy image J(i,j) in (39) will be assimilated to the intermediate reconstructed image, with a given degree of distortion (in our case, blurring) compared to the ideal image, I(i,j).

The gradually reconstructed images are denoted as follows, Ir1_2, Ir1_3, Ir1_4, Ir1_5, Ir1_6, and Ir1_7, where Ir1_2 is the image obtained by summing the first two components (LP and BP1), Ir1_3 is the image obtained by summing the first three components, and so on, while Ir1_7 is obtained by summing all seven components. In Table 3, the resulting values for the RMSE and PSNR, using Expressions (39) and (40), are displayed. Therefore, the PSNR obtained by adding up all component sub-band images is 21.79 dB. In future work, we will try to improve this figure by a better design and optimization of the 2D elliptical filter bank.

### 3.3. Application of Selective Directional Filtering in Oriented Straight Line Detection

The following set of simulations highlight the effect of very selective directional filtering on images containing oriented details. As is well known in image processing, the spectrum of a straight line from an image also has the shape of a straight line in the frequency plane $\left(\omega_{1},\omega_{2}\right)$, passing through the origin and orthogonal to the line direction.

**Figure 7:**
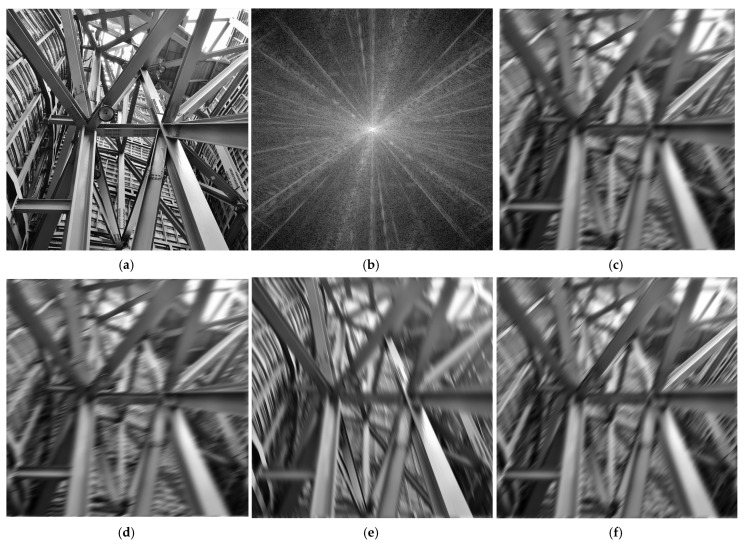

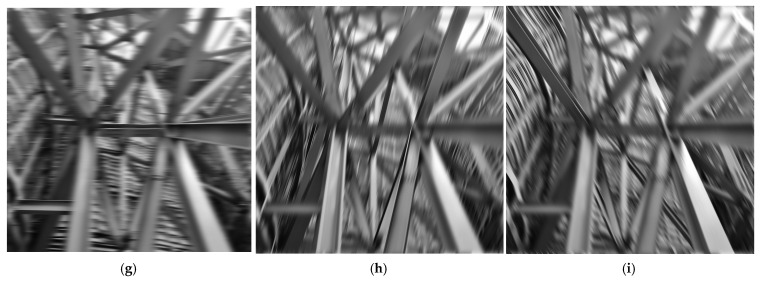
(**a**) Grayscale test image “steel structure”; (**b**) its logarithmic FFT spectrum magnitude; (**c**–**h**) directionally filtered output images for orientation angles: (**c**) φ=−π/6; (**d**) φ=−π/8; (**e**) φ=−π/10; (**f**) φ=−π/4; (**g**) φ=0; (**h**) φ=π/12; (**i**) φ=π/3.

Let us consider the image of size 690 × 690 pixels shown in Figure 7a, representing a structure of metallic beams, seen in perspective. This image is relevant for our directional filtering example, as it contains many straight lines oriented in various directions.

The logarithmic FFT spectrum of this image is displayed in Figure 7b. It has an interesting structure, consisting of fine straight lines of various orientations, corresponding to the various straight lines in the image. Taking into account the spectrum structure, the directional filtering can be easily understood. Thus, for a specified orientation angle of the directional filter, only the lines whose spectra overlap along the axis with the narrow filter characteristic will be preserved unaltered in the output image. The lines with other orientation will be more or less blurred. Lines with the spectrum perpendicular to the filter axis will be practically deleted due to blurring.

Applying a very selective Gaussian elliptical filter, like the ones shown in Figure 4, with various orientation angles (as specified in the figure), the filtered images in Figure 7c–g are obtained. For a specified orientation of the filter, different straight lines (various edges of metallic beams and other linear details) are outlined, while others are more or less affected by blurring, depending on the angle of orientation.

In the following section, an efficient implementation at the system level is proposed.

## 4. Polyphase Implementation of Designed 2D Elliptical FIR Filters

In the following, an efficient implementation is proposed at the system level for the designed 2D FIR elliptically shaped filters, relying on a polyphase decomposition of a 2D filtering operation with a convolution kernel of a relatively large size. In order to handle convolution with such a large kernel, block processing [21,22] and a polyphase decomposition approach are used in this work.

Firstly, using sub-expression sharing techniques, we obtain a fast algorithm that computes four outputs with a three-tap FIR filter for a 3 × 4 filtering operation, with a minimum number of multiplications. It has been shown that the minimal filtering algorithm for computing m outputs with an r-tap FIR filter, which we call F(m, r), requires µ(F(m, r)) = m + r − 1 multiplications [24]. Then, we can nest minimal 1D algorithms to form minimal 2D algorithms for computing 4 × 4 outputs with an 3 × 3 kernel filter [25]. These require the design of an efficient 2D filtering algorithm with a 3 × 3 kernel, but which can compute 4 × 4 output samples in parallel. In this section, a set of matrix equations describing the proposed novel filtering algorithm is presented. The obtained algorithm has been verified in MATLAB, version R2017a.

Thus, two partial output images, denoted as $Y_{0}$ and $Y_{1}$, given by Equations (41) and (42), are obtained:

(41) $$\begin{array}{l} Y_{0}=\left[\begin{array}{cccc} A_{0} & A_{0} & A_{0} & A_{0}\\ O_{4\times 7} & A_{0} & -A_{0} & O_{4\times 7}\\ O_{4\times 7} & A_{0} & A_{0} & -A_{0}\\ O_{4\times 7} & A_{0} & -A_{0} & O_{4\times 7} \end{array}\right]\times diag\left(\left[\begin{array}{ccc} A_{1} & O_{7\times 3} & O_{7\times 3}\\ 1/4\cdot A_{1} & 1/4\cdot A_{1} & 1/4\cdot A_{1}\\ 1/4\cdot A_{1} & -1/4\cdot A_{1} & 1/4\cdot A_{1}\\ 1/2\cdot A_{1} & O_{7\times 3} & -1/2\cdot A_{1} \end{array}\right]\times rev(H^{T})\right)\times \\ \;\times \left[\begin{array}{cccccc} A_{2} & O_{7\times 6} & O_{7\times 6} & O_{7\times 6} & -A_{2} & O_{7\times 6}\\ O_{7\times 6} & A_{2} & A_{2} & A_{2} & A_{2} & O_{7\times 6}\\ O_{7\times 6} & -A_{2} & A_{2} & -A_{2} & A_{2} & O_{7\times 6}\\ O_{7\times 6} & -A_{2} & -A_{2} & A_{2} & A_{2} & O_{7\times 6} \end{array}\right]\times X_{2D} \end{array}$$

(42) $$\begin{array}{l} Y_{1}=\left[\begin{array}{ccc} A_{0} & O_{4\times 7} & O_{4\times 7}\\ A_{0} & A_{0} & O_{4\times 7}\\ -A_{0} & O_{4\times 7} & O_{4\times 7}\\ -A_{0} & -A_{0} & A_{0} \end{array}\right]\times diag\left(\left[\begin{array}{ccc} 1/2\cdot A_{1} & 1/2\cdot A_{1} & -1/2\cdot A_{1}\\ O_{7\times 3} & 1/2\cdot A_{1} & O_{7\times 3}\\ O_{7\times 3} & O_{7\times 3} & A_{1} \end{array}\right]\times rev(H^{T})\right)\times \\ \;\times \left[\begin{array}{cccccc} O_{7\times 6} & A_{2} & O_{7\times 6} & -A_{2} & O_{7\times 6} & O_{7\times 6}\\ O_{7\times 6} & -A_{2} & A_{2} & A_{2} & -A_{2} & O_{7\times 6}\\ O_{7\times 6} & -A_{2} & O_{7\times 6} & O_{7\times 6} & O_{7\times 6} & A_{2} \end{array}\right]\times X_{2D} \end{array}$$

In the above equations, $rev(H^{T})$ means the reversed version of vector $H^{T}$, in which its elements are flipped (mirrored) horizontally.

Using the previous partial results, $Y_{0}$ and $Y_{1}$, we next compute the output vector containing 16 samples of the 2D filtering operation using the following equation:

(43) $$Y=Y_{0}+Y_{1}$$

where the output samples are given by

(44) $$Y={\left[\begin{array}{cccccccccccccccc} Y_{00} & Y_{01} & Y_{02} & Y_{03} & Y_{10} & Y_{11} & Y_{12} & Y_{13} & Y_{20} & Y_{21} & Y_{22} & Y_{23} & Y_{30} & Y_{31} & Y_{32} & Y_{33} \end{array}\right]}^{T}$$

and the block matrices $A_{0}$, $A_{1}$ and $A_{2}$ have the following structure:

(45) $$A_{0}=\left[\begin{array}{ccccccc} 1 & 1 & 1 & 1 & 1 & 0 & 0\\ 0 & 1 & -1 & 0 & 1 & 1 & 0\\ 0 & 1 & 1 & -1 & -1 & 0 & 0\\ 0 & 1 & -1 & 0 & -1 & -1 & 1 \end{array}\right]\;\;\;\;\;\;\;\;\;A_{1}=\left[\begin{array}{ccc} 1 & 0 & 0\\ 1/4 & 1/4 & 1/4\\ 1/4 & -1/4 & 1/4\\ 1/2 & 0 & -1/2\\ 1/2 & 1/2 & -1/2\\ 0 & 1/2 & 0\\ 0 & 0 & 1 \end{array}\right]\;\;\;\;\;\;\;\;A_{2}=\left[\begin{array}{cccccc} 1 & 0 & 0 & 0 & -1 & 0\\ 0 & 1 & 1 & 1 & 1 & 0\\ 0 & -1 & 1 & -1 & 1 & 0\\ 0 & -1 & -1 & 1 & 1 & 0\\ 0 & 1 & 0 & -1 & 0 & 0\\ 0 & -1 & 1 & 1 & -1 & 0\\ 0 & -1 & 0 & 0 & 0 & 1 \end{array}\right]$$

and $O_{4\times 7}$, $O_{7\times 3}$, $O_{7\times 6}$ are zero matrices of size 4×7, 7×3, and 7×6, respectively.

Also, we have vector *H* of the form

(46) $$H={\left[\begin{array}{ccccccccc} h_{00} & h_{01} & h_{02} & h_{10} & h_{11} & h_{12} & h_{20} & h_{21} & h_{22} \end{array}\right]}^{T}$$

while input vector $X_{2D}$ has the following structure:

(47) $$X_{2D}={\left[\begin{array}{ccccccccccccccc} x_{00} & x_{01} & \ldots & x_{05} & x_{10} & x_{11} & \ldots & x_{15} & \ldots & x_{50} & x_{51} & x_{52} & x_{53} & x_{54} & x_{55} \end{array}\right]}^{T}$$

The above algorithm for a 2D FIR filtering operation has been derived in order to reduce the number of operations, especially multiplications, due to the fact that there are redundant operations in the direct implementation of a 2D FIR filter. Using a new 2D algorithm, we significantly reduced the number of the multiplications not only compared with the direct implementation of a 2D FIR filter but also as compared with a recently proposed algorithm [23]. Using an efficient subexpression sharing technique, it was possible to obtain an increased reduction in the arithmetic complexity especially of the number of multiplications, as can be seen in the above algorithm for a 2D FIR filtering operation.

Using a block polyphase decomposition and the previous fast algorithm, we can obtain an efficient algorithm for the computation of the 2D FIR filter with a large kernel. In order to extend 2D filtering operations at a larger kernel, a block processing approach using polyphase decomposition will be used. In order to obtain 2D polyphase implementation using the proposed 2D algorithm from Equations (41)–(43), a decimation factor of 3 was used. With this value, a 12 × 12 input matrix was obtained.

For an easier understanding of our method, our discussion was restricted to a particular case where the kernel matrix was 9 × 9 and the input matrix was 12 × 12. However, the obtained results can be easily extended for our designed filter matrix. In order to use our previous algorithm for a 2D filter, a decimation factor of 3 was applied, both for the kernel matrix and for the input matrix.

Equations (41)–(43) can now be reformulated for a polyphase block implementation as will be shown below. Vector *h* is replaced with a new vector, defined as

(48) $$h_{2D}={\left[\begin{array}{ccccccccc} H_{22} & H_{21} & H_{20} & H_{12} & H_{11} & H_{10} & H_{02} & H_{01} & H_{00} \end{array}\right]}^{T}$$

Vectors $H_{00}$, $H_{01}$, $H_{02}$, $H_{10}$, $H_{11}$, $H_{12}$, $H_{20}$, $H_{21}$ and $H_{22}$ for the case where the kernel matrix is 9 × 9 and the input matrix is 12 × 12 are defined below.

First, vector $H_{i,j}$ has the following generic, parameterized form:

(49) $$H_{i,j}=\left[\begin{array}{ccccccccc} h_{6+i,6+j} & h_{6+i,3+j} & h_{6+i,j} & h_{3+i,6+j} & h_{3+i,3+j} & h_{3+i,j} & h_{i,6+j} & h_{i,3+j} & h_{i,j} \end{array}\right]$$

from which we derive nine vectors obtained for *i*, *j* = 0, 1, 2; for instance, the following vectors are derived for the indicated indices *i* and *j*:

(50) $$H_{00}=\left[\begin{array}{ccccccccc} h_{6,6} & h_{6,3} & h_{6,0} & h_{3,7} & h_{3,3} & h_{3,0} & h_{0,6} & h_{0,3} & h_{0,0} \end{array}\right]$$

(51) $$H_{01}=\left[\begin{array}{ccccccccc} h_{6,7} & h_{6,4} & h_{6,1} & h_{3,7} & h_{3,4} & h_{3,1} & h_{0,7} & h_{0,4} & h_{0,1} \end{array}\right]$$

(52) $$H_{21}=\left[\begin{array}{ccccccccc} h_{8,7} & h_{8,4} & h_{8,1} & h_{5,7} & h_{5,4} & h_{5,1} & h_{2,7} & h_{2,4} & h_{2,1} \end{array}\right]$$

Applying decimation with 3 instead of the kernel matrix of size 9 × 9, we obtain nine matrices of size 3 × 3. For example, for $H_{01}^{T}$ using decimation with factor 3, the following 3 × 3 block matrix was obtained:

(53) $$H_{21}^{\prime }=\left[\begin{array}{ccc} h_{21} & h_{24} & h_{27}\\ h_{51} & h_{54} & h_{57}\\ h_{81} & h_{84} & h_{87} \end{array}\right]$$

We take the rows of matrix $H_{21}^{\prime }$ and concatenate them, and then by reversing the order of the elements, vector $H_{21}$ is obtained:

(54) $$H_{21}=\left[\begin{array}{ccccccccc} h_{8,7} & h_{8,4} & h_{8,1} & h_{5,7} & h_{5,4} & h_{5,1} & h_{2,7} & h_{2,4} & h_{2,1} \end{array}\right]$$

Vector $x_{2D}$ is also replaced with vector $X_{2D}$, defined as

(55) $$X_{2D}={\left[\begin{array}{ccccccccccccccc} X_{00} & X_{01} & \ldots & X_{05} & X_{10} & X_{11} & \ldots & X_{15} & \ldots & X_{50} & X_{51} & X_{52} & X_{53} & X_{54} & X_{55} \end{array}\right]}^{T}$$

Vectors $X_{i,j}$, have the following generic, parameterized form:

(56) $$X_{i,j}=\left[\begin{array}{ccccccccc} x_{6+i,6+j} & x_{6+i,3+j} & x_{6+i,j} & x_{3+i,6+j} & x_{3+i,3+j} & x_{3+i,j} & x_{i,6+j} & x_{i,3+j} & x_{i,j} \end{array}\right]$$

From this general form, by giving successive values (0, 1, 2,…,5) to indices *i* and *j*, all 36 vectors are derived. For instance, the following vectors are obtained for the indicated index values:

(57) $$X_{12}=\left[\begin{array}{ccccccccc} x_{7,8} & x_{7,5} & x_{7,2} & x_{4,8} & x_{4,5} & x_{4,2} & x_{1,8} & x_{1,5} & x_{1,2} \end{array}\right]\;(i=1,j=2)$$

(58) $$X_{23}=\left[\begin{array}{ccccccccc} x_{8,9} & x_{8,6} & x_{8,3} & x_{5,9} & x_{5,6} & x_{5,3} & x_{2,9} & x_{2,6} & x_{2,3} \end{array}\right]\;(i=2,j=3)$$

(59) $$X_{31}=\left[\begin{array}{ccccccccc} x_{9,7} & x_{9,4} & x_{9,1} & x_{6,7} & x_{6,4} & x_{6,1} & x_{3,7} & x_{3,4} & x_{3,1} \end{array}\right]\;(i=3,j=1)$$

(60) $$X_{45}=\left[\begin{array}{ccccccccc} x_{10,11} & x_{10,8} & x_{10,5} & x_{7,11} & x_{7,8} & x_{7,5} & x_{4,1} & x_{4,8} & x_{4,5} \end{array}\right]\;(i=4,j=5)$$

Applying decimation with a factor 3, instead of the input matrix of 12 × 12, we obtain nine matrices of size 3 × 3. As an example, for vector *X*_21_, using decimation with a factor of 3, the following 3 × 3 block matrix results:


(61) $$X\prime_{21}=\left[\begin{array}{ccc} x_{21} & x_{24} & x_{27}\\ x_{51} & x_{54} & x_{57}\\ x_{81} & x_{84} & x_{87} \end{array}\right]$$

At this step, we take the rows of matrix $X\prime_{21}$, concatenate them, and then by reversing the order of the elements, the following matrix $X_{21}$ is obtained:

(62) $$X_{21}=\left[\begin{array}{ccccccccc} x_{8,7} & x_{8,4} & x_{8,1} & x_{5,7} & x_{5,4} & x_{5,1} & x_{2,7} & x_{2,4} & x_{2,1} \end{array}\right]$$

Thus, we decomposed a 2D FIR filtering operation with a 9 × 9 kernel into 49 inner products, as shown below.

In the preprocessing stage, it is first necessary to obtain, from a 12 × 12 input matrix, matrix $X\prime_{ij}$ of size 3 × 3, using a decimation operation. Then, by taking the rows of matrix $X\prime_{ij}$, vectors $X_{ij}$ will be obtained, as has been shown previously for matrix $X\prime_{21}$.

Using polyphase decomposition and the obtained equations for the 2D FIR filter (41)–(43), the following equations are used for the polyphase implementation of our 2D FIR filter with a 9 × 9 kernel:

(63) $$Y_{0}=\left[\begin{array}{cccc} A_{0} & A_{0} & A_{0} & A_{0}\\ O_{4\times 7} & A_{0} & -A_{0} & O_{4\times 7}\\ O_{4\times 7} & A_{0} & A_{0} & -A_{0}\\ O_{4\times 7} & A_{0} & -A_{0} & O_{4\times 7} \end{array}\right]\times d\prime_{2D}\;\;\;\;Y_{1}=\left[\begin{array}{ccc} A_{0} & O_{4\times 7} & O_{4\times 7}\\ A_{0} & A_{0} & O_{4\times 7}\\ -A_{0} & O_{4\times 7} & O_{4\times 7}\\ -A_{0} & -A_{0} & A_{0} \end{array}\right]\times d\prime \prime_{2D}$$

where $O_{4\times 7}$ is a null matrix of size 4 × 7, $Y=Y_{0}+Y_{1}$, and also, we have the following vectors:

(64) $$d\prime_{2D}=\left[\begin{array}{cccccccccccccccccccccccccccc} d_{00} & d_{01} & d_{02} & d_{03} & d_{04} & d_{05} & d_{06} & d_{10} & d_{11} & d_{12} & d_{13} & d_{14} & d_{15} & d_{16} & d_{20} & d_{21} & d_{22} & d_{23} & d_{24} & d_{25} & d_{26} & d_{30} & d_{31} & d_{32} & d_{33} & d_{34} & d_{35} & d_{36} \end{array}\right]$$

(65) $$d\prime \prime_{2D}=\left[\begin{array}{ccccccccccccccccccccc} d_{40} & d_{41} & d_{42} & d_{43} & d_{44} & d_{45} & d_{46} & d_{50} & d_{51} & d_{52} & d_{53} & d_{54} & d_{55} & d_{56} & d_{60} & d_{61} & d_{62} & d_{63} & d_{64} & d_{65} & d_{66} \end{array}\right]$$

In order to obtain the final output samples, a postprocessing stage is required, in which the partial results $Y_{0}$ and $Y_{1}$ are added.

To determine the elements of vector $d\prime_{2D}$, we first compute

(66) $$H_{C1}=\left[\begin{array}{ccc} B_{1} & O_{63\times 27} & O_{63\times 27}\\ 1/4\cdot B_{1} & 1/4\cdot B_{1} & 1/4\cdot B_{1}\\ 1/4\cdot B_{1} & -1/4\cdot B_{1} & 1/4\cdot B_{1}\\ 1/2\cdot B_{1} & O_{63\times 27} & -1/2\cdot B_{1} \end{array}\right]\times {\left[\begin{array}{ccccccccc} H_{22} & H_{21} & H_{20} & H_{12} & H_{11} & H_{10} & H_{02} & H_{01} & H_{00} \end{array}\right]}^{T}$$

(67) $$X_{C1}=\left[\begin{array}{cccccc} B_{2} & O_{63\times 54} & O_{63\times 54} & O_{63\times 54} & -B_{2} & O_{63\times 54}\\ O_{63\times 54} & B_{2} & B_{2} & B_{2} & B_{2} & O_{63\times 54}\\ O_{63\times 54} & -B_{2} & B_{2} & -B_{2} & B_{2} & O_{63\times 54}\\ O_{63\times 54} & -B_{2} & -B_{2} & B_{2} & B_{2} & O_{63\times 54} \end{array}\right]\times X_{2D}$$

Then, vectors $H_{C1}$ and $X_{C1}$ are split into smaller sections of nine elements, and the elements of auxiliary vector $d\prime_{2D}$ are computed using an inner product operation, as follows:

(68) $$\begin{array}{l} d_{00}=H_{C1}(1:9)•X_{C1}(1:9)\\ ..........................................\\ d_{06}=H_{C1}(55:63)•X_{C1}(55:63)\\ d_{10}=H_{C1}(64:72)•X_{C1}(64:72)\\ ..........................................\\ d_{16}=H_{C1}(118:126)•X_{C1}(118:126)\\ d_{20}=H_{C1}(127:135)•X_{C1}(127:135)\\ ..........................................\\ d_{26}=H_{C1}(181:189)•X_{C1}(181:189)\\ d_{30}=H_{C1}(190:198)•X_{C1}(190:198)\\ ..........................................\\ d_{36}=H_{C1}(244:252)•X_{C1}(244:252) \end{array}$$

where “•” denotes the inner product operation.

In a similar way, the elements of auxiliary vector $d\prime \prime_{2D}$ are computed by splitting the two vectors, $H_{C1}$ and $X_{C1}$, into smaller sections of nine elements, using an inner product operation. Vectors $H_{C2}$ and $X_{C2}$ result from the following equations:

(69) $$H_{C2}=\left[\begin{array}{ccc} 1/2\cdot B_{1} & 1/2\cdot B_{1} & -1/2\cdot B_{1}\\ O_{63\times 27} & 1/2\cdot B_{1} & O_{63\times 27}\\ O_{63\times 27} & O_{63\times 27} & B_{1} \end{array}\right]\times {\left[\begin{array}{ccccccccc} H_{22} & H_{21} & H_{20} & H_{12} & H_{11} & H_{10} & H_{2} & H_{01} & H_{00} \end{array}\right]}^{T}$$

(70) $$X_{C2}=\left[\begin{array}{cccccc} O_{63\times 54} & B_{2} & O_{63\times 54} & -B_{2} & O_{63\times 54} & O_{63\times 54}\\ O_{63\times 54} & -B_{2} & B_{2} & B_{2} & -B_{2} & O_{63\times 54}\\ O_{63\times 54} & -B_{2} & O_{63\times 54} & O_{63\times 54} & O_{63\times 54} & B_{2} \end{array}\right]\times X_{2D}$$

with $B_{1}=A_{1}⊗I_{9}$ and $B_{2}=A_{2}⊗I_{9}$, where matrix $I_{9}$ is the 9×9 identity matrix (with ones on the main diagonal and zeros elsewhere), with the structure

(71) $$I_{9}=\left[\begin{array}{ccccccccc} 1 & 0 & 0 & 0 & 0 & 0 & 0 & 0 & 0\\ 0 & 1 & 0 & 0 & 0 & 0 & 0 & 0 & 0\\ 0 & 0 & 1 & 0 & 0 & 0 & 0 & 0 & 0\\ 0 & 0 & 0 & 1 & 0 & 0 & 0 & 0 & 0\\ 0 & 0 & 0 & 0 & 1 & 0 & 0 & 0 & 0\\ 0 & 0 & 0 & 0 & 0 & 1 & 0 & 0 & 0\\ 0 & 0 & 0 & 0 & 0 & 0 & 1 & 0 & 0\\ 0 & 0 & 0 & 0 & 0 & 0 & 0 & 1 & 0\\ 0 & 0 & 0 & 0 & 0 & 0 & 0 & 0 & 1 \end{array}\right]$$

and $O_{63\times 54}$ and $O_{63\times 27}$ are zero matrices of sizes 63 × 54 and 63 × 27, respectively. Then, we can compute, as before, the intermediate values:

(72) $$\begin{array}{l} d_{40}=H_{C2}(1:9)•X_{C2}(1:9)\\ ..........................................\\ d_{46}=H_{C2}(55:63)•X_{C2}(55:63)\\ d_{50}=H_{C2}(64:72)•X_{C2}(64:72)\\ ..........................................\\ d_{56}=H_{C2}(118:126)•X_{C2}(118:126)\\ d_{60}=H_{C2}(127:135)•X_{C2}(127:135)\\ ..........................................\\ d_{66}=H_{C2}(181:189)•X_{C2}(181:189) \end{array}$$

Also, as can be seen from Equations (68) and (72), in order to introduce polyphase decomposition, it was necessary to introduce a post-processing block that performs a segmentation of vectors $H_{C1}$, $H_{C2}$, $X_{C1}$, and $X_{C2}$ in segments of nine elements that are used in 49 inner product operations.

The two auxilliary vectors, $d\prime_{2D}$ and $d\prime \prime_{2D}$, were introduced to facilitate the reformulation of the algorithm for 2D FIR filtering, with polyphase implementation.

This polyphase algorithm was developed for a 2D FIR filtering operation, with the aim to reduce computational complexity, since it is well known that there are redundant operations in the direct implementation of 2D convolution. In the direct approach, there are, for instance, overlapping input data blocks. For this reason, eliminating redundancy as much as possible is essential for reducing the number of arithmetic operations. The novel approach proposed here, using a subexpression sharing technique, allows for an efficient implementation structure with a high degree of parallelism and therefore an increased speed of operation.

## 5. Discussion

The proposed design method for 2D elliptically shaped filters is entirely analytical, without using any global numerical optimization techniques. Analytical design techniques lead to closed-form and parametric filters, with adjustable, tunable frequency responses. To the best of the authors’ knowledge, analytical designs for 2D FIR elliptical filter banks have not been previously studied. For this reason, a rigorous comparison in terms of performance with other elliptical filters found in the literature, like [14,15,16,17,18], is quite difficult to make since the design approaches, filter characteristics and purposes are very different.

For instance, paper [18] approaches the design and optimization of the performance of 2D FIR filters with circular and elliptical symmetry. However, it differs significantly from our analytical design method since it uses a global optimization approach, based on a particular, very efficient meta-heuristic algorithm, namely the Salp-Swarm optimization algorithm. As in every numerical optimization algorithm, it starts from an initial set of random parameters, regarded as a population, which is updated over a number of iterations until some fitness condition is met, specifically an imposed maximum and average ripple in the pass-band and stop-band. The 2D FIR filter kernel resulting from the optimization process cannot be, in general, decomposed and the frequency response cannot be factored, as in our analytical method.

For the elliptically shaped filter, the authors have chosen some fixed semi-axes values (the aspect ratio) and the ellipse axes are parallel with the frequency plane axes. Also, in [18], only maximally flat, low-pass circular and elliptical filters with a wide band have been designed. By comparison, in our paper, we have designed narrow-band elliptically shaped filters based on a Gaussian prototype (of the LP, BP and HP type, forming an uniform filter bank) and they are oriented at a specified angle in the frequency plane. In particular, we have designed an elliptical Gaussian filter bank based on a uniform 1D prototype with specified peak frequencies and bandwidths.

However, the filters can be roughly compared regarding the maximum ripple in the stop-band. Due to the specific Gaussian shape, our filters have no ripple in the pass-band. The stop-band ripple of the derived 2D filters would be rather difficult to measure exactly, but in principle, its maximum values are approximately the ones of their prototypes, given in Table 1 from Section 2. It can be noticed that these maximum stop-band ripple values are lower than the ones of the elliptical filters designed in [18].

The elliptically shaped filter bank designed in this work can be regarded as a continuation of the work [23], in which circular 2D FIR filter banks were designed. However, the design technique is more complex for elliptical filters, the expression of the frequency transformation being more complicated than in the circular case. As with circular filter banks, we have analyzed a test image, decomposing and then reconstructing it relatively accurate from its sub-band components. This suggests the eventual possibility of using such filters in image coding. Referring to existing works on related topics, other analytical techniques for designing 2D elliptically shaped filters of the IIR type were previously proposed in [24,25].

For this application, we chose the Gaussian filter as a prototype for the 2D filter bank, due to its well-known advantages. Among others, it can be easily and efficiently approximated and can also be scaled on the frequency axis to modify its selectivity. Because its frequency response is of a zero phase, the frequency components of an image will not be phase-shifted, and thus, the filter will not produce image distortions. The resulting filters have accurate shapes, with negligible distortions.

The proposed solution was based on a new efficient algorithm for a 2D FIR filtering operation with lower arithmetic complexity for a smaller kernel of 3 × 3, which was then extended at a larger kernel size of 30 × 30 using a polyphase decomposition of the given 2D filtering operation and block filtering.

In order to facilitate the reformulation of the algorithm for 2D FIR filtering for a polyphase implementation, we introduced some auxiliary vectors. So, it was possible to extend the proposed algorithm 2D algorithm for a significantly larger size.

Using the proposed algorithm we have significantly reduced the complexity of the algorithm while maintaining the same throughput of 16 output samples at a time, as in [23]. Thus, instead of 100 inner products as in [23], we have only 49 inner products in the proposed algorithm. In the simpler case that was used, where the kernel size was 9 × 9, each inner product involved nine multiplications. Thus, instead of 900 multiplications as in [23], we now have only 49 × 9 = 441 multiplications. This advantage is even more important in the case of hardware implementation.

## 6. Conclusions

The proposed design technique for 2D elliptically shaped filter banks is fully analytical, without using any complicated numerical optimization algorithms. It is mainly based on a 1D to 2D frequency transformation which is especially developed using various approximations and is applied to the 1D prototype, yielding directly a factored 2D frequency response, which may be useful in a sequential implementation. Equivalently, the overall filter kernel results were directly decomposed as a convolution product of smaller matrices. The major advantage of this novel design approach is that the resulting filter is parametric. The filter specifications (the selectivity and orientation angle) appear explicitly in the filter kernel and the filter is adjustable. Thus, for any given specifications, the filter is determined directly, and there is no need to resume every time the entire design process. This approach leads to filters with accurate shapes and low distortions. As shown through simulations, the uniform elliptical filter bank decomposes an image into sub-band components, with increasing frequency content and decreasing energy.

Then, by adding these sub-band images one by one, starting from the low-frequency component, the initial image is gradually reconstructed. By summing all the sub-band images, the reconstructed image is visually quite close to the original test image, the finest details being quite clearly visible. The reconstruction quality was evaluated numerically using measures such as the RMSE and PSNR.

Regarding the implementation part, the proposed solution based on the polyphase and block filtering approach is very efficient and leads to a filter structure with reduced arithmetical complexity. This is true compared not only to the direct filtering approach (which can be very complex for large filter kernels and large images) but also to the most recent similar solution presented in [23].

In a future continuation of this work, we will study more rigorously the design of 2D elliptical filter banks and their applications in image analysis, in particular, focusing on the improvement of image reconstruction quality.

## Figures and Tables

**Table 1:** The maximum stop-band ripple for the components of the filter bank.

Filter Type	LP	BP1	BP2	BP3	BP4	BP5	HP
Maximum ripple in stop-band	0.00298	0.00459	0.00361	0.002421	0.004601	0.00441	0.00321

**Table 2:** Relative energies (in %) for the 7 images resulting from the output of the uniform elliptical filter bank.

Relative Energy	ER1	ER2	ER3	ER4	ER5	ER6	ER7
Value (%)	53.364	13.891	8.346	6.2154	4.5651	3.6136	3.1323

**Table 3:** Values of RMSE and PSNR (in dB) for the 6 partially reconstructed images.

Partially Reconstructed Image	Ir1_2	Ir1_3	Ir1_4	Ir1_5	Ir1_6	Ir1_7
RMSE	102.1	58.15	37.59	27.83	23.49	20.75
PSNR (dB)	7.96	12.84	16.63	19.24	20.71	21.79

## Data Availability

The original contributions presented in this study are included in the article. Further inquiries can be directed to the corresponding author.
